# Removal of Basic Violet 14 from aqueous solution using sulphuric acid activated materials

**DOI:** 10.1186/s40064-016-2294-2

**Published:** 2016-05-17

**Authors:** S. Suresh

**Affiliations:** Department of Chemistry, St. Joseph University in Tanzania, Arusha Campus, Arusha, Tanzania

**Keywords:** Sulphuric acid activated materials, Adsorption, Dye removal, Equilibrium, Kinetics

## Abstract

In this study the adsorption of Basic Violet, 14 from aqueous solution onto sulphuric acid activated materials prepared from *Calophyllum inophyllum* (CS) and *Theobroma cacao* (TS) shells were investigated. The experimental data were analysed by Langmuir, Freundlich and Temkin isotherm models. The results showed that CS has a superior adsorption capacity compared to the TS. The adsorption capacity was found to be 1416.43 mg/g for CS and 980.39 mg/g for TS. The kinetic data results at different concentrations were analysed using pseudo first-order and pseudo-second order model. Boyd plot indicates that the dye adsorption onto CS and TS is controlled by film diffusion. The adsorbents were characterised by scanning electron microscopy. The materials used in this study were economical waste products and hence can be an attractive alternative to costlier adsorbents for dye removal in industrial wastewater treatment processes.

## Background

Colour is an important characteristic of effluent and it leads to serious environmental threat. The highly coloured dyes affect the water bodies by inhibiting sunlight penetration and hence affecting the photosynthetic activity. These highly coloured dyes are extensively used for colouring in industries like textile, paper, leather and cosmetic industries. Dyes and pigments are highly toxic, carcinogenic and mutagenic (Dutta 1994; Yagub et al. 2014). The worldwide dye consumption in textile industry is more than 10^7^ kg/year that is mainly used in fabrics (Ahamed et al. 2007).

As dyes have a complex structure and synthetic origin, it is difficult to decolourise and various treatment methods have different efficiency in treating dye waste water. There are different treatment methods to decolourise dye waste water like coagulation (Orfao et al. 2006), Photocatalytic degradation (Sun et al. 2008), electrochemical, degradation (Fan et al. 2008), chemical oxidation, ozonation and coagulation (Arslan 2001; Kim et al. 2005). However, these processes are costly and cannot be effectively used for a wide range of dye wastewater.

Adsorption using low cost adsorbents is widely used, since it is one of the most effective methods for dye removal from wastewaters because of their unique properties in adsorption of both cationic and anionic dyes. The advantages of adsorption process are simplicity in operation, inexpensive compared to other separation methods, insensitivity to toxic substances and no sludge formation (Waranusantigul et al. 2003).

In treatment of colored effluents different low-cost adsorbents have been investigated at the laboratory scale for effective treatment with different degrees of success (Bhattacharyya and Sharma 2005). Some of the low cost adsorbents are, waste pea shells (Khan et al. 2014), water chestnut peel (Khan et al. 2013), bamboo sawdust (Khan and Nazir 2015), Curcuma angustifolia scales (Maiyalagan et al. 2014), Curry tree seed (Suresh et al. 2011a) Curry tree stem (Suresh et al. 2011b), etc. Still there is a need for effective adsorbents in dye wastewater treatment.

In this study the paper compares the ability of two sulphuric acid activated materials for removing Basic Violet 14 from aqueous solution using the shells of *Calophyllum inophyllum* (CS) and *Theobroma cacao* (TS) shells.

## Experimental

### Materials

**Fig. 1:**
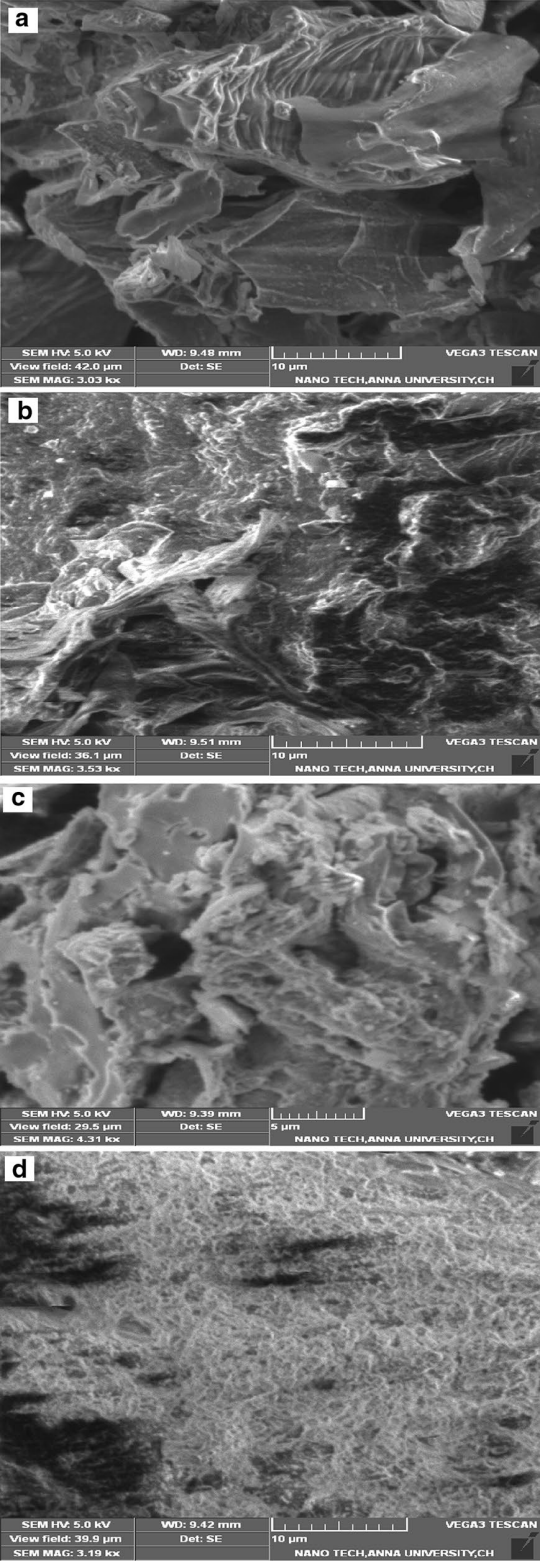
SEM micrograph of CS **a** before dye adsorption, **b** after dye adsorption and TS adsorbent material **c** before dye adsorption, **d** after dye adsorption

Basic Violet 14 is a triaminotriphenylmethane dye (CI 42,510; Molecular weight: 337.85 g mol^−1^; Molecular Formula: C_20_H_19_N_3_·HCl; Maximum Wavelength: 540 nm. The molecular structure of Basic Violet 14 is shown in Fig. 1. The dye is inflammable and is used in coloring of textile and leather materials. The dye on ingestion may cause nausea, vomiting, diarrhea and the inhalation of dye causes irritation to respiratory tract. In humans and animals, its toxicity includes carcinogenic and mutagenic effects (Littlefield et al. 1985).

### Preparation of adsorbent

The raw material *Calophyllum inophyllum* (CS) shell and *Theobroma cacao* (TS) shells were collected and it is washed with water to remove the dirt, dust and other surface impurities. The washed shells were dried for 24 h. The dried shells were then soaked in 18N∙H_2_SO_4_ (1:2, w/v) and kept in oven at 120 °C for 12 h. This is done to activate the carbonaceous material by chemical activation. The product is washed several times with distilled water and soaked in 1 % sodium bicarbonate solution for 12 h to remove any residual acid and kept in oven at 110 °C for 12 h. The acid treated biomass adsorbent, thus obtained is crushed and sieved to uniform particle size using ASTM standard sieve (Mesh No. 100). The adsorbents thus obtained were labeled as CS and TS.

### Batch adsorption experiments

Adsorption experiments were carried out with CS and TS at a varying dye concentration with a fixed adsorbent dose. To study the efficiency to remove Basic Violet 14 from aqueous solution and to find the isotherm constants, experiments were conducted with, 50 mL of various concentrations of dye solutions in a conical flask with fixed adsorbent dosage. Experiments were carried out in the natural pH of the dye solution at room temperature. This mixture was agitated on a mechanical shaker at a constant speed for about 2 h. The dye solutions, after agitation were separated from the adsorbent and the equilibrium dye concentrations were determined spectrophotometrically by measuring the absorbance changes at the wavelength of maximum absorbance (540 nm). Kinetic experiments were carried out using a mechanical stirrer in the concentration range of 260–380 mg/L. The percentage dye removal was accessed using the formula.

1 $${\text{Percentage}}\;{\text{dye}}\;{\text{removal}} = \frac{{{\text{C}}_{\text{i}} - {\text{C}}_{\text{f}} \, }}{{{\text{C}}_{\text{i}} }} \times 100$$

The amount adsorbed at equilibrium q_e_ (mg/g) was calculated by

2 $${\text{Amount}}\;{\text{adsorbed}}\;\left( {q_{\text{e}} } \right) = {\text{C}}_{\text{i}} - {\text{C}}_{\text{f}} \times \frac{\text{V}}{\text{M}}$$

where *C*_i_ and *C*_f_ are the liquid phase concentrations of the dye at initial and final concentrations (mg/L) respectively. *M* the mass (g) of adsorbents and *V* is the volume of dye solution (L).

## Results and discussion

### Characterization of adsorbent

The surface morphologies of the sulphuric acid activated adsorbents CS and TS were analyzed by scanning electron microscroscope (SEM). The surface of the adsorbents CS and TS prior to adsorption process and after the adsorption was shown in Fig. 1a–d. It is clear, that the adsorbents have a rough morphology with considerable porous nature where suitable conditions exist for the dye to be trapped and adsorbed into the adsorbent. The SEM image of CS shows a good morphology compared to TS for adsorption. It is evident from Fig. 1b, that the surface of CS is covered by a layer of Basic Violet 14 and significant changes were observed due to dye adsorption. In TS as a result of entrapment of the dye into the adsorbent a homogeneously dye adhered surface can be observed in Fig. 1d.

**Fig. 2:**
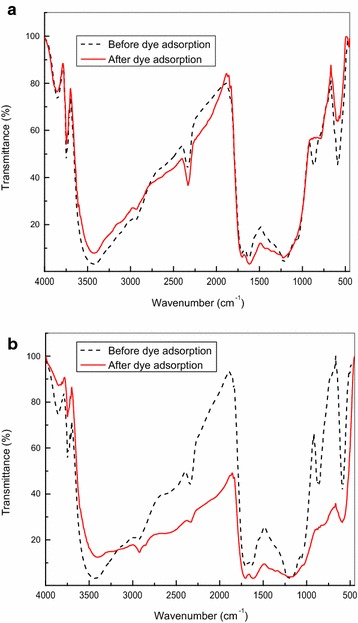
FT-IR spectra of **a** CS, **b** TS adsorbent material

The functional groups on CS and TS were identified using FT-IR and the results of the characterized samples were shown in Fig. 2a, b. The band at 3421 cm^−1^ in the adsorbent CS represented O–H stretching vibration of alcoholic groups. The band at 2336 cm^−1^ corresponds to the N–H stretching (Hameed and El-Khaiary 2008). The band at 1699 cm^−1^ is attributed to the C=O stretching of carboxylate anion (Minamisawa et al. 2004). The band at 1221 cm^−1^ is assigned to S=O stretching. The band at 860 cm^−1^ is seen due the presence of ${-}{\text{SO}}_{3}^{ - }$ group (Kannan 2014; Figueira et al. 1999) and this band disappears when CS is loaded with the dye. The functional group in this region seems to participate in dye binding. The band at 590 cm^−1^ is due to C-S stretching. TS shows absorption band at 3423 cm^−1^ due to O–H stretching of hydroxyl groups. The band at 2930 cm^−1^ in TS is due to a weak alkyl C–H stretching. The band at 1700 cm^−1^ is due to C=O stretching. The bands at 1623 cm^−1^ and 1200 cm^−1^ corresponds to C=C stretching and S=O stretching. The bands at 861 cm^−1^ and 871 cm^−1^ due to S=O indicates the presence of –SO3̶ group, is observed to disappear when TS is loaded with the dye and hence the functional group in this region participates in dye binding. The presence of the band at 590 cm^−1^ in CS and 589 cm^−1^ in TS is due to C–S stretching and this band is reduced in band height after adsorption and hence, it appears that this group involves in adsorption. The surface of the sulphuric acid activated adsorbent surface of CS and TS has polar functional group present in it that provides anion exchange capacity for the dye. As the reaction proceeds, in CS and TS the negatively charged surface functional group interacts strongly with the dye molecule to facilitate the ion exchange process, and hence provides good adsorption capacity for the adsorbents.

### Effect of adsorbent dosage

To find the effect of adsorbent dosage on to the adsorbate, a fixed adsorbate concentration of 260 mg/L and a constant volume (50 mL) is taken, keeping all other experimental conditions constant. It is observed that the amount of Basic Violet 14 adsorbed decreases as the concentration of the adsorbent increases. Thus, it can be observed that maximum dye removal occurs at 10 mg to 30 mg in both CS and TS and then decreased with increase in adsorbent mass.

**Fig. 3:**
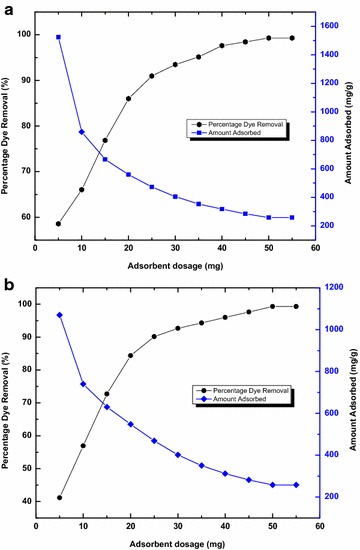
Effect of adsorbent dosage **a** CS, **b** TS adsorbent (C_0_ = 260 mg/L; V = 0.05 L)

From Fig. 3, it is observed that for an increase in CS and TS adsorption from 5 to 55 mg the dye uptake decreases from 1523 to 258 mg/g for CS and 1069 to 258 mg/g for TS. The dye adsorption increases with the increase in adsorbent doses and after a particular limit the adsorption slowly decreases and becomes constant for CS and TS. The increase in percentage colour removal with adsorbent dosage can be attributed to the availability of more adsorption sites (Garg et al. 2003). Another reason for the decrease in adsorption capacity with increase in adsorbent mass is an aggregation/agglomeration of adsorbent particles at higher concentrations. It is observed that dye removal by CS and TS is effective at a low adsorbent dosage for the removal of Basic Violet 14 from aqueous solution.

### Adsorption isotherms

The linear form of three isotherms namely Langmuir and Freundlich and Temkin (Hameed and Daud 2008) were used to analyse the isotherm data. The adsorption isotherm is important from theoretical and practical point of view. The linear form of the equation is given as

3 $${\text{C}}_{\text{e}} /{\text{q}}_{\text{e}} = 1/{\text{Q}}_{\hbox{max} } {\text{K}}_{\text{L}} + {\text{C}}_{\text{e}} /{\text{Q}}_{\hbox{max} }$$

4 $$\log q_{\text{e}} = 1/n\log C_{\text{e}} + \log {\text{K}}_{\text{F}}$$

5 $$q_{\text{e}} = B\ln A + B\ln C_{\text{e}}$$

**Fig. 4:**
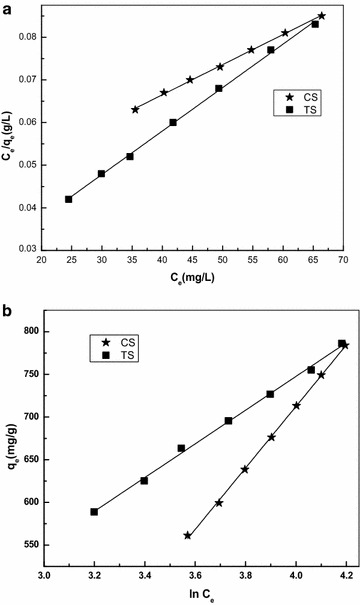
**a** Langmuir isotherm, **b** Temkin isotherm for CS and TS adsorbent

The values of *Q*_m_ and *K*_L_ were calculated from the slope and intercept of the linear plot (Fig. 4a). The better R^2^ value for Langmuir isotherm indicates the applicability of this model for CS and TS. The applicability of the Langmuir isotherm for both CS and TS suggests the monolayer coverage of adsorbate on the surface of adsorbents (Langmuir 1918). The isotherm parameters obtained using the linear form of Eq. (3) is given in Table 1. The R_L_ value observed indicates the type of isotherm to be favourable (0 < *R*_L_ < 1), linear (*R*_L_ = 1), unfavourable (*R*_L_ > 1) or irreversible *R*_L_ = 0. The value *K*_L_ is the Langmuir constant and *C*_0_ is the highest initial dye concentration (mg/L). The value of R_L_ is found to be in the range of 0.1761 to 0.1278 for CS and 0.0612 to 0.0417 for TS,

**Table 1:** Langmuir, Freundlich and Temkin constants for CS and TS

	CS	TS
Langmuir constants		
*Q*_m_ (mg/g)	1416.43	980.39
*K*_L_ (L/mg)	0.018	0.059
*R* ^2^	0.9987	0.9984
*R*_*L*_	0.1761–0.1278	0.0612–0.0427
Freundlich constants		
*K*_F_ (mg/g)	81.69	238.39
*n*	1.8508	3.5075
*R* ^2^	0.9978	0.9940
Temkin constants		
*K*_T_ (L/mg)	0.1322	0.8050
*B*_1_	360.23	197.55
*R* ^2^	0.9983	0.9981

6 $$R_{\text{L}} = 1/(1 + K_{\text{L}} C_{0} )$$

From Table 1, *Q*_m_, the maximum monolayer adsorption capacity of CS is found to be 1416.43 mg/g and for TS is found to be 980.39 mg/g. The applicability of the linear form of the Langmuir model to CS and TS, proved by the high correlation coefficients *R*^2^ > 0.998 suggests that the Langmuir isotherm provides a good model of the sorption system.

The Freundlich constants K_F_ and n can be calculated from the slope and intercept of the linear plot with log q_e_ versus logC_e_. The magnitude of the component ‘n’ gives an indication of the favourability of adsorption process and K_F_ is the constant related to the adsorption capacity.

According to Treybal (1980), it has been shown that n > 1, represents favorable adsorption. The n value was found to be 1.85 for CS and 3.51 for TS which indicates favourable adsorption. 1/n indicates the adsorption intensity of dye onto the adsorbent or surface heterogeneity, becoming more heterogeneous as its value gets closer to 0. The isotherm constants K_f_ and n were calculated from the linear form of the model and the value of K_f_, n, and the Correlation Coefficients are given in the Table 1.

The linear form of the Temkin equation is used to analyze the adsorption data and it is observed that the Temkin isotherm fitted well for CS and TS. The Temkin isotherm constants were calculated from the plot of q_e_ versus ln C_e_ (Fig. 4b) and are given in the Table 1. In CS and TS the Temkin isotherm fitted well with a high correlation coefficient. The isotherm data’s were well represented by the Langmuir, and Temkin isotherm with R^2^ values fitting in the following series, Langmuir > Temkin > Freundlich. In CS and TS the R^2^ values were found to be high (>0.99) for all the three isotherms studied, hence, it can be concluded that both monolayer and heterogenous surface conditions exists in the present study. Since CS and TS is observed to have good adsorption capacity and hence it can be used as an effective, low cost adsorbent as an alternative material to commercial activated carbon in the removal of dyes from aqueous solution.

### Kinetics

In order to analyse the adsorption process for pseudo first order and pseudo second order model (Hameed and Daud 2008) the kinetic equations were used.

7 $${\text{Log}}\left( {{\text{q}}_{\text{e}} - {\text{q}}_{\text{t}} } \right) = \log {\text{q}}_{\text{e}} {-}{\text{k}}_{1} /2.303{\text{t}}$$

8 $${\text{t}}/{\text{q}}_{\text{t}} = 1/{\text{k}}_{2} {\text{q}}_{\text{t}}^{2} + {\text{t}}/{\text{q}}_{\text{t}}$$

The kinetic study of CS and TS at different initial dye concentration was carried out and the kinetic data were analyzed using pseudo first order and pseudo second order model. The kinetic parameters give important information for designing and modeling the adsorption process. It is observed that the adsorption of dye on the adsorbent surface takes place by an initial rapid binding of the dye molecule on the adsorbent.

The correlation coefficient (R^2^) value from Table 2 shows that the pseudo second order model provides the best fit for the adsorption of CS and TS. The correlation coefficient of 0.9973 to 0.9983 for CS indicates that it is a good correlation for pseudo second order model and hence this model is suitable to provide the best fit when compared to the pseudo first order model. The correlation coefficient in TS is >0.997 for pseudo second order model and <0.996 in pseudo first order model. This confirms that pseudo second order model provides the best fit in both CS and TS.

**Table 2:** Kinetic model values for the adsorption of Basic Violet 14 on to CS and TS

Adsorbent	Concentration (mg/L)	q_e(exp)_ (mg/g)	Pseudo first order values			Pseudo second order values			
			q_e(Calc)_ (mg/g)	k_1_ (min^−1^)	R^2^	q_e(Calc)_ (mg g^−1^)	k_2_ (g mg^−1^ min^−1^)	h (mg g^−1^min^−1^)	R^2^
CS	260	133.71	123.19	7.83 × 10^−2^	0.9959	142.85	7.96 × 10^−4^	16.24	0.9980
	300	169.23	127.84	7.18 × 10^−2^	0.9950	169.49	8.27 × 10^−4^	23.75	0.9983
	340	191.29	125.14	6.52 × 10^−2^	0.9915	196.07	8.42 × 10^−4^	32.37	0.9973
	380	185.93	151.01	6.70 × 10^−2^	0.9953	199.60	5.74 × 10^−4^	22.87	0.9979
TS	260	133.71	74.03	10.13 × 10^−2^	0.9904	140.84	2.14 × 10^−3^	42.45	0.9985
	300	145.91	92.44	9.70 × 10^−2^	0.9968	147.05	1.69 × 10^−3^	36.54	0.9985
	340	167.81	104.47	9.44 × 10^−2^	0.9952	172.41	1.38 × 10^−3^	41.02	0.9978
	380	149.19	73.65	9.05 × 10^−2^	0.9961	153.84	2.00 × 10^−3^	47.33	0.9980

A Plot of log(q_e_ − q_t_) versus time for CS and TS enables to calculate the rate constant k_1_ and from the slope and intercept of the plot q_e(pred)_ can be calculated. The adsorption kinetics of Basic Violet 14 from aqueous solution by CS and TS is studied and the influence of various operating parameters on the adsorption process is evaluated. It is observed that the dye uptake was very rapid and the saturation time was found to be 60 min in CS and 40 min in TS.

A plot of t/qt versus t enables to calculate the rate constant k_2_ that is used to calculate the initial sorption rate h as follows

9 $${\text{h}} = {\text{k}}_{2} {\text{q}}_{\text{e}}^{2}$$

**Fig. 5:**
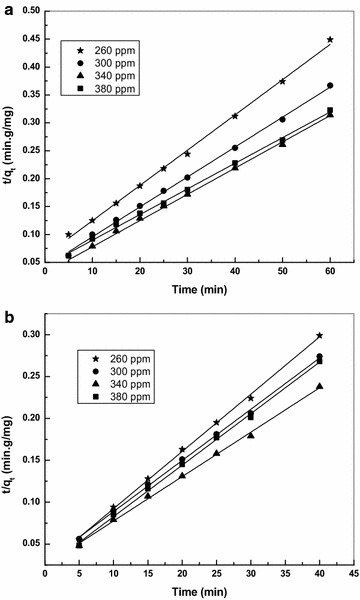
Pseudo second order Kinetics plot for the adsorption of Basic violet 14 onto **a** CS and **b** TS adsorbent

Now, the initial sorption rate h, rate constant k_2_ and q_e(calc)_ for CS and TS can be obtained from the plot of t/q_t_ versus t (Fig. 5). The q_e_ values calculated from the pseudo-second order model system is in better agreement with the experimental q_e_ values for CS and TS. The second order rate constant k_2_ was found to increase and decrease Basic Violet 14 concentration in CS. A similar trend was observed in the literature for the increase and decrease in k_2_ with an increase in solute concentration (Ahmad and Rahman 2011). However, in most of the adsorption systems, there exists a trend between the initial dye concentrations and k_2_ with a decreasing k_2_ value with the increase in initial dye concentration. A similar trend was observed in TS, with a decrease in k_2_ for an increase in initial dye concentration (Ho 2004). The correlation coefficient (R^2^) of the linear plot is very high (>0.997) for all the concentrations studied in pseudo second order model. The material surface has negatively charged surface functional group which interacts strongly with the cationic dye influencing the adsorption process. The values of kinetic constants and q_e_ of Basic Violet 14 adsorption onto CS and TS are given in Table 2.

The Table 3 gives the adsorption capacity of some low cost adsorbents used for the adsorption of dyes along with CS and TS. It is clear that in this study the adsorption of the basic dye by the sulphuric acid activated materials prepared from CS and TS is good when compared with some of the adsorbents already reported for the adsorption of basic dyes from aqueous solution.

**Table 3:** Adsorption capacities of different low cost adsorbents for dye removal from aqueous solution

Adsorbent	Dyes	Adsorption capacity (mg/g)	References
Neem sawdust	Basic Violet 10	2.35	Khattri and Singh (2000)
Sugarcane dust	Basic Violet 10	13.90	Ho et al. (2005)
Coir pith	Basic Violet 10	2.56	Namasivayam et al. (2001)
Activated sludge biomass	Basic Violet 3	113.6	Chu and Chen (2002)
Sugarcane dust	Basic Violet 3	3.79	Khattri and Singh (1999)
Deoiled Soya	Basic Violet 14	12.03	Gupta et al. (2008)
Bottom ash	Basic Violet 14	6.39	Gupta et al. (2008)
*Curcuma angustifolia* Scales	Basic Violet 14	208.33	Maiyalagan et al. (2014)
*Calophyllum inophyllum* Shells	Basic Violet 14	1416.43	This work
*Theobroma cacao* Shells	Basic Violet 14	980.39	This work

### Adsorption mechanism

**Fig. 6:**
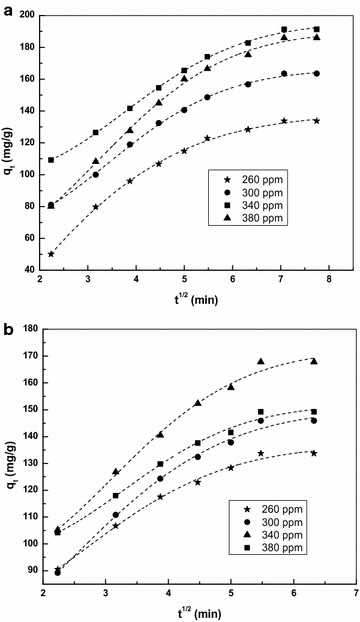
Intraparticle diffusion plot for the adsorption of Basic violet 14 onto **a** CS and **b** TS adsorbent

The adsorption kinetic data of CS and TS were analysed to identify the diffusion mechanism using the Intraparticle diffusion model proposed by Weber and Morris (Weber and Morris 1963)

10 $${\text{q}}_{\text{t}} = {\text{k}}_{\text{p}} {\text{t}}^{1/2} + {\text{C}}$$

where k_p_ is the intraparticle diffusion rate constant and C is the intercept that is obtained from the slope of the plot of q_t_ versus t^1/2^ (Fig. 6). The k_p_ for CS and TS were found to be in the range of 95.92 to 130.82 for CS and 98.53 to 115.12 for TS. The two phases in the CS and TS intraparticle diffusion plot represents an initial surface adsorption in the reaction and then the next phase is intraparticle diffusion. In the plot of q_t_ versus t^1/2^ for CS and TS if the straight line passes through the origin, then intraparticle diffusion is the only rate controlling step, or if it does not pass through the origin, then surface adsorption is the rate controlling step. In the present study for both CS and TS the plot does not pass through the origin, hence surface adsorption is the rate controlling step. The intercept C value in Table 4 discusses about the thickness of the boundary layer. The increase in the constant C for CS and TS indicates internal mass transfer and hence the increase in thickness of the boundary layer.

**Table 4:** Intraparticle diffusion coefficient and diffusion coefficient (D_i_) for CS and TS

Adsorbent	Concentration (mg/L)	K_ip_ (mg/g min^0.5^)	D_i_ (cm^2^/s)
CS	260	95.92	9.16 × 10^−11^
	300	112.18	8.41 × 10^−11^
	340	130.82	7.64 × 10^−11^
	380	116.80	7.85 × 10^−11^
TS	260	98.53	1.18 × 10^−10^
	300	100.37	1.13 × 10^−10^
	340	115.12	1.11 × 10^−10^
	380	109.29	1.06 × 10^−10^

**Fig. 7:**
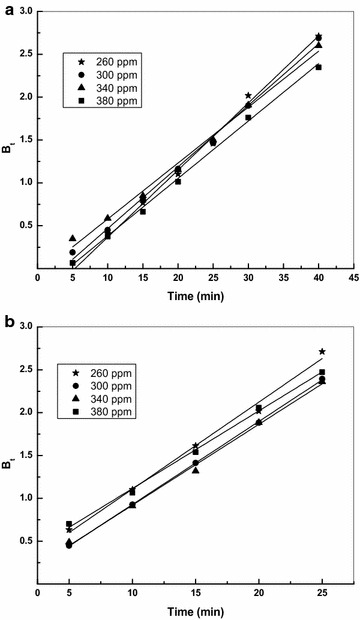
Boyd plot for the adsorption of Basic violet 14 onto **a** CS and **b** TS adsorbent

Three types of mechanism are involved in the adsorption process like film diffusion, particle diffusion and the adsorption of the solute molecules on the interior surface of the adsorbent (Chingobe et al. 2006). Since, the third step is fast and negligible it is necessary to distinguish between film and particle diffusion. Therefore Boyd model is used to study the actual slow step using the expression (Boyd et al. 1947)

11 $${\text{Bt}} = - 0.4977 - \ln (1 - {\text{F}})$$

where, F represents the fraction of dye adsorbed at any time t, and Bt is a mathematical function of F. The B_t_ versus time plot (Fig. 7) For CS and TS distinguishes film diffusion and particle diffusion. If the plots are linear and pass through the origin, then the slowest step in the adsorption process is the particle diffusion and if the linear plot does not pass through the origin then film diffusion controls the adsorption process. In this study the plots for CS and TS were found to be linear, at all concentrations and does not pass through the origin, confirming surface adsorption as the rate limiting step. The dye cation interacts strongly with the negatively charged surface functional group in the sulphuric acid activated materials resulting in effective adsorption. The B values were used to calculate the effective diffusion coefficient, D_i_ (m^2^/s) using the following relationship

12 $${\text{B}} = \frac{{\pi^{2} {\text{D}}_{\text{i}} }}{{{\text{r}}^{2} }}$$

The B values were calculated for CS and TS and the results are shown in the Table 4.

## Conclusion

Basic Violet 14 adsorbs effectively in the surface of the two sulphuric acid activated adsorbents CS and TS and equilibrium is attained in 60 min for CS and 40 min for TS. The isotherm data prove monolayer adsorption for CS and TS and the adsorption capacity was found to be 1416.43 mg/g for CS and 980.39 mg/g for TS. The kinetic study proves that the pseudo second order model provides the best fit for both the adsorbents. Film diffusion process is involved in the adsorption of Basic Violet 14 onto CS and TS. The comparative studies on the adsorption of prepared activated carbons CS and TS onto Basic Violet 14 showed a higher percentage of dye removal for CS. This study shows that the sulphuric acid activated adsorbent CS and TS can be used as an effective material in the adsorption of Basic Violet 14 from aqueous solution.

## References

[CR1] Ahamed AA, Hameed BH, Aziz N (2007). Adsorption of direct dye on palm ash. Kinetic and equilibrium modeling. J Hazard Mater.

[CR2] Ahmad MA, Rahman NK (2011). Equilibrium, kinetics and thermodynamic of Remazol Brilliant orange 3R dye adsorption on coffee husk based activated carbon. Chem Eng J.

[CR3] Arslan I (2001). Treatability of a simulated disperse dye-bath by ferrous iron coagulation, ozonation, and ferrous iron-catalyzed ozonation. J Hazard Mater.

[CR4] Bhattacharyya KG, Sharma A (2005). Kinetics and thermodynamics of methylene blue adsorption on Neem (*Azadirachta indica*) leaf powder. Dyes Pigm.

[CR5] Boyd GE, Adamson AW, Myers LS (1947). The exchange adsorption of ions from aqueous solution by organic zeolites II. Kinetics. J Am Chem Soc.

[CR6] Chingobe P, Saha B, Wakeman RJ (2006). Sorption of atrazine on conventional and surface modified activated carbons. J Colloid Interface Sci.

[CR7] Chu HC, Chen KM (2002). Reuse of activated sludge biomass: removal of basic dyes from wastewater by biomass. Process Biochem.

[CR8] Dutta PK (1994). An overview of textile pollution and its remedy. Indian J Environ Prot.

[CR9] Fan L, Zhou Y, Yang W, Chen G, Yang F (2008). Electrochemical degradation of aqueous solution of Amaranth azo dye on ACF under potentiostatic model. Dyes Pigm.

[CR10] Figueira MM, Volesky B, Mathieu H (1999). Instrumental analysis study of iron species biosorption by *Sargassum* biomass. Environ Sci Technol.

[CR11] Garg VK, Gupta R, Yadav AB, Kumar R (2003). Dye removal from aqueous solution by adsorption on treated saw dust. Bioresour Technol.

[CR12] Gupta VK, Mittal A, Gajbe V, Mittal J (2008). Adsorption of basic fuchsin using waste materials-bottom ash and deoiled soya-as adsorbents. J Colloid Interface Sci.

[CR13] Hameed BH, Daud FBM (2008). Adsorption studies of basic dye on activated carbon derived from agricultural waste: *Hevea brasiliensis* seed coat. Chem Eng J.

[CR14] Hameed BH, El-Khaiary MI (2008). Removal of Basic dye from aqueous medium using a novel agricultural waste material: pumpkin seed hull. J Hazard Mater.

[CR15] Ho YS (2004). Pseudo-isotherms using a second order kinetic expression constant. Adsorption.

[CR16] Ho YS, Chiu WT, Wang CC (2005). Regression analysis for the sorption isotherm of basic dyes on sugarcane dust. Bioresour Technol.

[CR17] Kannan TA (2014). FTIR and EDS analysis of the seaweeds *Sargassum wightii* (brown algae) and *Gracilaria corticata* (red algae). Int J Curr Microbiol Appl Sci.

[CR18] Khan TA, Nazir M (2015). Enhanced adsorptive removal of a model acid dye bromothymol blue from aqueous solution using magnetic chitosan–bamboo sawdust composite: batch and column studies. Environ Prog Sustain Energy.

[CR19] Khan TA, Nazir M, Khan EA (2013). Adsorptive removal of rhodamine B from textile wastewater using water chestnut (*Trapa natans* L.) peel: adsorption dynamics and kinetic studies. Toxicol Environ Chem.

[CR20] Khan TA, Rahman R, Ali I, Khan EA, Mukhlif AA (2014). Removal of malachite green from aqueous solution using waste pea shells as low cost adsorbent–adsorption isotherms and dynamics. Toxicol Environ Chem.

[CR21] Khattri SD, Singh MK (1999). Colour removal from dye waste water using sugarcane dust as an adsorbent. Adsorpt Sci Technol.

[CR22] Khattri SD, Singh MK (2000). Colour removal from synthetic dye wastewater using a biosorbent. Water Air Soil Pollut.

[CR23] Kim TH, Park C, Yang J, Kim S (2005). Comparison of disperse and reactive dye removal by chemical coagulation and Fenton oxidation. J Hazard Mater.

[CR24] Langmuir I (1918). The adsorption of gases on plane surfaces of glass, mica, and platinum. J Am Chem Soc.

[CR25] Littlefield NA, Blackwell BN, Hewitt CC, Gaylor DW (1985). Chronic toxicity and carcinogenicity studies of Gentian violet in mice. Fundam Appl Toxicol.

[CR26] Maiyalagan T, Suresh S, Wilfred Sugumar R (2014). A low cost adsorbent prepared from *Curcuma angustifolia* scales for removal of Basic Violet 14 from aqueous solution. Indian J Chem Technol.

[CR27] Minamisawa M, Minamisawa H, Yoshida S, Takai N (2004). Adsorption behavior of heavy metals on biomaterials. J Agric Food Chem.

[CR28] Namasivayam C, Dinesh Kumar M, Selvi K, Ashruffunissa Begum R, Vanathi T, Yamuna RT (2001). ‘Waste’ Coir pith-a potential biomass for the treatment of dyeing wastewaters. Biomass Bioenergy.

[CR29] Orfao JJM, Silva AIM, Pereira JCV, Barata SA, Fonseca IM, Faria PCC, Pireira MFR (2006). Adsorption of reactive dyes on chemically modified activated carbons—influence of pH. J Colloid Interface Sci.

[CR30] Sun J, Qiao I, Sun S, Wang G (2008). Photocatalytic degradation of orange G on nitrogen-doped TiO_2_ catalysts under visible light and sunlight irradiation. J Hazard Mater.

[CR31] Suresh S, Wilfred Sugumar R, Maiyalagan T (2011). Adsorption of Acid red 18 from aqueous solution onto activated carbon prepared from *Murraya Koenigii* (curry tree) seeds. Asian J Chem.

[CR32] Suresh S, Wilfred Sugumar R, Maiyalagan T (2011). Equilibrium and Kinetic studies on the adsorption of Methylene blue from aqueous solution onto activated carbon prepared from *Murraya koenigii* (curry tree) stems. Asian J Chem.

[CR33] Treybal RE (1980). Mass transfer operations.

[CR34] Waranusantigul P, Pokethitiyook P, Kruatrachue M, Upatham ES (2003). Kinetics of basic dye (Methylene blue) biosorption by giant duckweed (*spirodelapolyrrhiza*). Environ Pollut.

[CR35] Weber WJ, Morris JC (1963). Kinetics of adsorption on carbon from solution. J Sanit Eng Div.

[CR36] Yagub MT, Sen TK, Afroze S, Ang HM (2014). Dye and its removal from aqueous solution by adsorption: a review. Adv Colloid Interface Sci.

